# Four-channel display and encryption by near-field reflection on nanoprinting metasurface

**DOI:** 10.1515/nanoph-2022-0216

**Published:** 2022-06-14

**Authors:** Yue Cao, Lili Tang, Jiaqi Li, Chengkuo Lee, Zheng-Gao Dong

**Affiliations:** School of Physics, Southeast University, Nanjing 211189, China; Department of Electrical and Computer Engineering, National University of Singapore, Singapore 117583, Singapore; Center for Intelligent Sensors and MEMS(CISM), National University of Singapore, Singapore 117542, Singapore

**Keywords:** four-channel display, metasurface, multichannel, near-field reflection

## Abstract

Multichannel metasurfaces become one of the most significant development trends, as they exhibit versatile manipulation abilities on electromagnetic fields and provide a promising approach to constitute compact devices with various complex functions, especially in optical encryption due to its capabilities of multichannel, high complexity, and high concealment. However, the existent multichannel metasurfaces based optical encryption technology can only realize two channels in the near-field, or perform three channels in near- and far-field. In this paper, a four-channel display metasurface used to encrypt information by three optical parameters as security keys is firstly proposed and experimentally demonstrated, which is different from the previous three-channel metasurface combined nanoprinting and hologram in near- and far-field. The novel design strategy of the four-channel metasurface can effectively enhance the information capacity and increase the difficulty of leaks without causing manufacturing challenges and additional costs. In addition, the simulation and experimental results demonstrate that the designed metasurface with four independent channels can separately display distinguishable nanoprinting images under decoding keys of special optical parameters. The proposed four-channel display metasurface with advantages of high capacity and ultracompactness will pave a way for multichannel applications in nano display, information storage, optical anticounterfeiting, and other relevant fields.

## 1 Introduction

Traditional encryption methods are difficult to distinguish between true and false [1], and easy to be detected or copied by lawless persons, such as intaglio [2], traditional printing technologies [3], chemical-based inks [4], laser security label [5], latent images [6], and conventional optical anticounterfeiting based on interference and diffraction [7], [8], [9]. With the advent of the information age, the demand for security of our personal information is becoming increasingly important, and new security methods to encrypt the information or cope with the continuous attack of fraud are paramount [10, 11]. Among the varieties of encryption methods adopted in anti-counterfeiting markets, optical encryption technologies, especially at the subwavelength scales, have stood out because of their excellent ability in controlling the numerous degrees of freedom of light [12], [13], [14], such as the amplitude [15], polarization [16], phase [17, 18], and angular momentum [19], which can generate various visual effects for anticounterfeiting productions. Optical encryption technologies can benefit from different combinations of the characteristics of light, which would generate innovative ideas for multifunctional encryption and decryption of information [20], [21], [22], [23].

Metasurfaces, rapidly developed ultrathin optical elements in recent decades, exhibit exceptional ability in manipulating amplitude, polarization, and phase of incident light at the level of subwavelength [24], [25], [26]. In the initial stage, nearly all metasurfaces used the nanostructural design method to manipulate only one character of light for a monofunctional device such as metagratings [27, 28], metalenses [29, 30], holograms [31, 32], and nanoprints [33], [34], [35], [36]. To enhance the information capacity, metasurfaces must possess multichannel characteristics by manipulating one or some of the degrees of freedom of incident light. Recently, various metasurfaces have been reported to realize multichannel/multifunctional optical devices [19, 37], [38], [39], [40], which provide the possibility of potential applications in information encryption, data storage, and optical communication. In particular, multichannel metasurfaces are proposed as candidates for data storage and information encryption owing to their superior ability in accelerating the development of miniaturization and integration as well as anticounterfeiting technology to protect information security. To date, metasurfaces have been manufactured to save an image with high resolution or more images in a single device, which is regarded as an effective approach to encrypt and decrypt information. In theory, the higher encryption and decryption security are, the more difficult is to steal information, which is a goal of high security to encrypt information. However, there are several recent reports about anticounterfeiting metasurfaces that only have up to three-channel or trifunctional by a polarization-controlled grayscale display and a phase-modulated hologram both in the near- and far-field [20, 41], [42], [43]. Furthermore, Zheng’s group manufactured an anticounterfeiting metasurface composed of a single-sized nanostructure [40], which can simultaneously realize a two-channel grayscale display in the near-field. This novel method provides an additional degree of freedom to enhance the encryption capacity in favor of high-security technology. To the best of our knowledge, the encryption metasurfaces generating more than three-channel for anticounterfeiting functions have yet to be reported [44, 45].

To increase/enhance the channel/capacity of encryption information, we propose an encoding method using amplitude, polarization, and wavelength as three degrees of freedom to design a four-channel display metasurface. It is different from the previous three-channel metasurface for near- and far-field combined functionalization in that our four-channel encryption metasurface is only operated in the near-field, which can display four encryption images by corresponding security keys. More importantly, our approach not only enhances information capacity but also simplifies the complexity of the imaging system without increasing any cost. Specifically, the simulation and experimental results demonstrate that four independent channels store four distinguishable nanoprinting images, respectively, which can only be decrypted with specific optical security keys. Our designed four-channel metasurface provides an effective strategy for potential applications, such as ultracompact display, high-density information storage, optical anticounterfeiting, and information hiding.

## 2 Results and discussion

**Figure 1:**
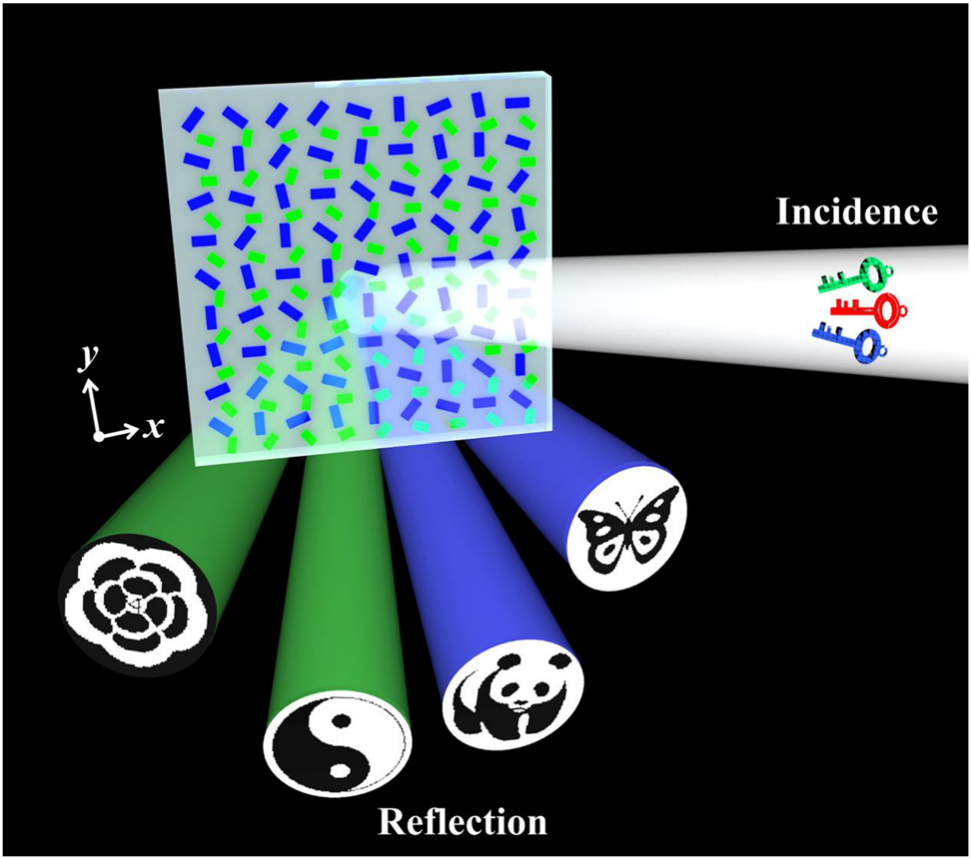
Schematic of four-channel display metasurface, which can separately store four distinguishable nanoprinting images and display them in the near-field reflection by specific security keys for independent channels. The metasurface is composed of two kinds of Ag nanorods with different orientations on a glass substrate. A normally incident linearly-polarized beam is used to display the hidden information encoded in the metasurface with optical parameters (amplitude, polarization, and wavelength) as specific security keys.

As shown in Figure 1, we propose a novel design scheme of a four-channel display metasurface consisting of monolayer nanorods, which can separately display four highly distinguishable nanoprinting images in independent channels under different optical illuminations as the security keys in the near-field. Here, the monolayer metasurface is consisting of two kinds of silver (Ag) nanorods with customized orientation angles on a silicon dioxide (SiO_2_) substrate. As shown in the scheme, there are three optical parameters (intensity, polarization, and wavelength) to control the encoded nanoprinting images, which are regarded as optical security keys for switching and displaying images. Specifically, four switchable images are exhibited in the near-field reflected light by changing the polarization and/or wavelength keys of normally incident illumination. More importantly, unlike other multichannel imaging metasurfaces to demonstrate multifunctional optical images using combination technologies of nanoprinting and holography, our multichannel imaging metasurface is significantly simple and effective to operate solely by nanoprinting (i.e., without a combined imaging system associated with complicated phase manipulation). Hence, our proposed metasurface with the four-channel displays of high information capacity for near-field compact imaging.

As is known, each Ag nanorod can reflect light in a way described by the Jones matrix, which has a close connection between the input and output field written as

(1) $$J_{\text{rod}}=\left[\begin{array}{cc} \cos\alpha & -\sin\alpha \\ \sin\alpha & \cos\alpha \end{array}\right]\left[\begin{array}{cc} A & 0\\ 0 & B \end{array}\right]\left[\begin{array}{cc} \cos\alpha & \sin\alpha \\ -\sin\alpha & \cos\alpha \end{array}\right]$$

where *α* is the orientation angle of the nanorod, *A* and *B* are the complex reflection coefficients of the nanostructure for lights linearly polarized along its fast and slow axes, respectively.

When the linearly polarized (LP) illumination with input intensity *I*_0_ passes through a polarizer with polarization direction *θ* and then is reflected by nanorods with orientation *α*, the output light *I* can be expressed as (for detailed derivations, see Section S1 in Supplemental Material).

(2) $$I=I_{0}\left[A^{2}cos^{2}\left(\alpha -\theta \right)+B^{2}\sin^{2}\left(\alpha -\theta \right)\right]$$

The proposed nanorods effectively manipulate copolarization reflection intensity, which can be regarded as an ideal polarizer for both long-axis and short-axis rod resonance modes. So, we can set as *A* = 1 and *B* = 0, the reflection intensity *I* is simply written as

(3) $$I=I_{0}cos^{2}\left(\alpha -\theta \right)$$

Because the polarization angle *θ* can be arbitrarily chosen, if we rotate the polarization direction *θ*′ based on the original polarization angle *θ*, the refection intensity *I* is written as

(4) $$I=I_{0}cos^{2}\left(\alpha -\theta -\theta^{\prime }\right)$$

The theoretical analysis demonstrates that the reflection intensity can be arbitrarily manipulated by controlling the LP direction of light with respect to the orientation angle of nanorods, which conveniently provides a switching parameter for encoding images.

**Figure 2:**
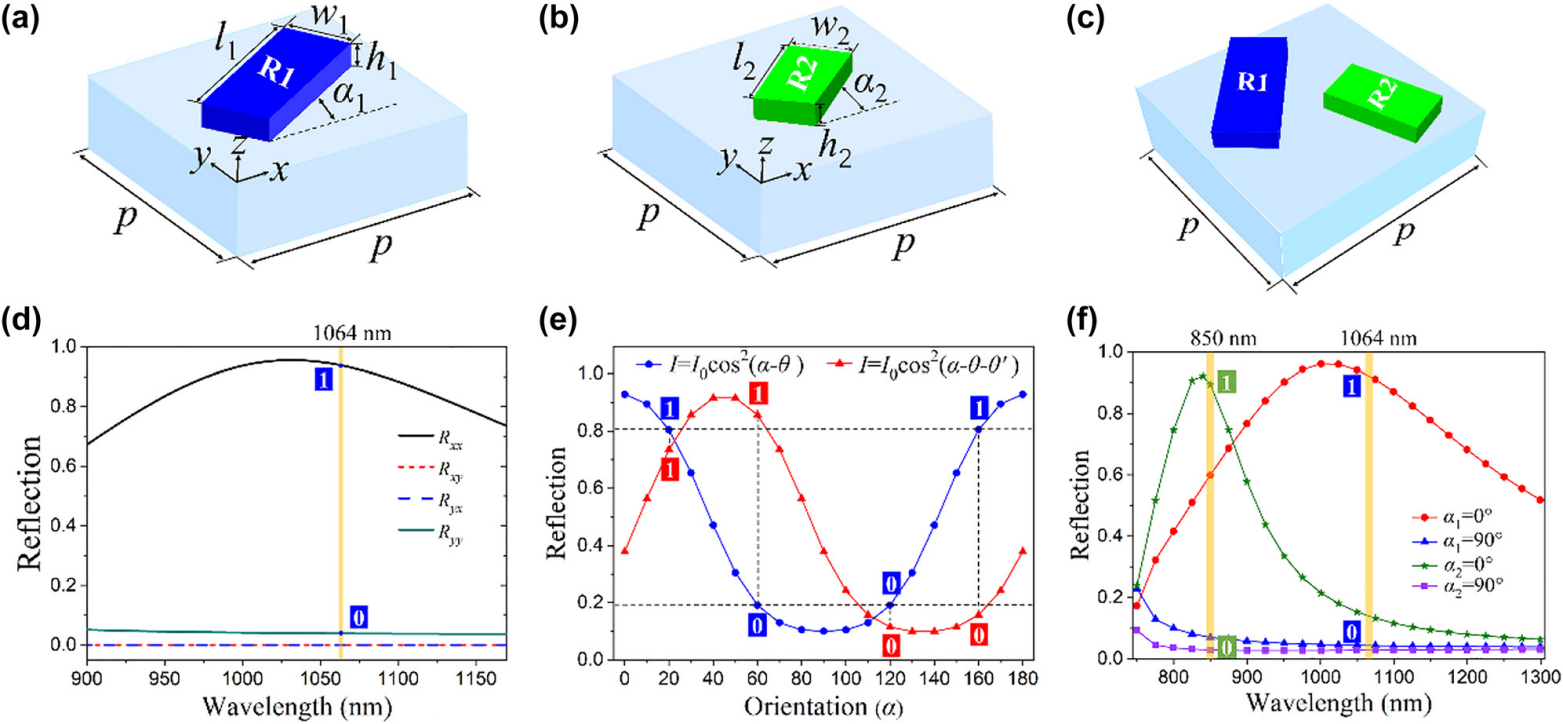
Schematics of unit cells and principle of encoded display metasurface. (a, b, c) Schematics of unit cells of single- and double-nanorod on SiO_2_ substrate. (d) Principle of reflection amplitude encoded single-channel display metasurface. The simulated reflection of different LP components from a metasurface composed of R1 nanorods with *α* = 0°. (e) Principle of polarization encoded multiplexed display metasurface. The reflection intensities in channels 1 and 2 satisfy *I* = *I*_0_cos^2^(*α*-*θ*) (blue curve) and *I* = *I*_0_cos^2^(*α*-*θ*-*θ′)* (red curve), respectively, where *α* is the orientation angle of each nanorod, *θ* is the original angle of polarizer for channel 1, *θ′* is the rotated angle of polarizer for channel 2. The curve shows that an identified orientation angle of nanorod can realize the intensity contrast of two polarization-encoded channels, for two polarization angles *θ* and *θ*-*θ′*, there are four orientation angles in the orientation interval [0, *π*], which can be regarded as the candidates for two-channel polarization encoded metasurface. (f) The principle of wavelength encoded display metasurface, which provides two channels at 1064 nm and 850 nm when the polarization angle along the long axis of the nanorod.

To realize the multiple channels, we design a metasurface composed of nanorods with different sizes and orientation angles. Figure 2a and b show schematics of the unit cell of R1 and R2 with blue and green colors, respectively. The parameters of two Ag nanorods on SiO_2_ substrate are as follows: R1 has length *l*_1_ = 330 nm, width *w*_1_ = 135 nm, height *h*_1_ = 100 nm; R2 has length *l*_2_ = 220 nm, width *w*_2_ = 120 nm, height *h*_2_ = 100 nm, the period of all metasurfaces *p* = 520 nm, and the orientation angles of R1 and R2 are marked as *α*_1_ and *α*_2_ with respect to the *x*-axis, respectively. Figure 2c exhibits a metasurface containing two kinds of nanorods (R1 and R2) of different sizes, which is regarded as a supercell meta-atom. Figure 2d shows the simulated reflection intensity of different LP components for the metasurface composed of R1 nanorods with *α* = 0°. The simulation results demonstrate that the proposed metasurface can obtain maximum *x*-polarization reflection for LP illumination along the *x*-axis around 1064 nm with a working efficiency greater than 90%, implying the excellent copolarization reflection capability of the nanorods for incident polarization component parallel to its long axis. Further, an encoded metasurface can be designed with two gray levels (i.e., bright and dark), corresponding to the marks signed as “1” and “0”, so-called amplitude encoded display metasurface. Figure 2e presents the principle of polarization encoded display metasurface based on the switching parameter of polarization angle of LP illumination, in which blue and red curves show the reflection intensities in dependence of the orientation angle of nanorods for two polarization channels, one is *θ* and another is *θ-θ’* (here *θ* = 0° and *θ’* = *π*/4). The simulated cosine-square curves indicate that there are two identical reflection intensities generated from two differently oriented nanorods for the blue curve, while the nanorods with the same orientation angle whose reflection intensities are extremely different under illumination *θ′* polarization difference. So, we can choose four nanorods with different orientation angles to generate two polarization encoded images into one metasurface. It is interesting to note that, the polarization direction, as an additional encoded key for multichannel display metasurface, does not influence the qualities of the encoded image as well as the fabrication feasibility as compared with a single-channel metasurface. Figure 2f shows the principle of wavelength encoded metasurface, which consists of R1 and R2 with different orientation angles. The reflection spectra of R1 and R2 with the orientation angles of 0° and 90° are plotted to show the designed operating wavelengths at 850 nm and 1064 nm. Here, each atom is regarded as an independent pixel; therefore the wavelength can be used as an encoded key for multichannel display metasurface. Depending on operating wavelengths, the reflection intensity can be encoded as “1” or “0”, which are associated with orientation angles of two sizes of nanorods. However, it can be found that the reflection intensity of R1 has reached about 0.5 at 850 nm, which implies that it could bring some crosstalk for wavelength encoded two-channel imaging. To reduce the crosstalk, the reflection intensity of additional nanorod R3 exceeding 0.9 at 780 nm (as shown in Figure S1) is adopted, meanwhile, the reflection intensity of R1 is less than 0.2 at 780 nm, which demonstrates that the crosstalk of two-wavelength channels for 1064 nm and 780 nm is small enough to be eliminated. Experimentally, R2 was fabricated due to the limitations of experimental conditions, and a detailed explanation is shown in the following section.

**Figure 3:**
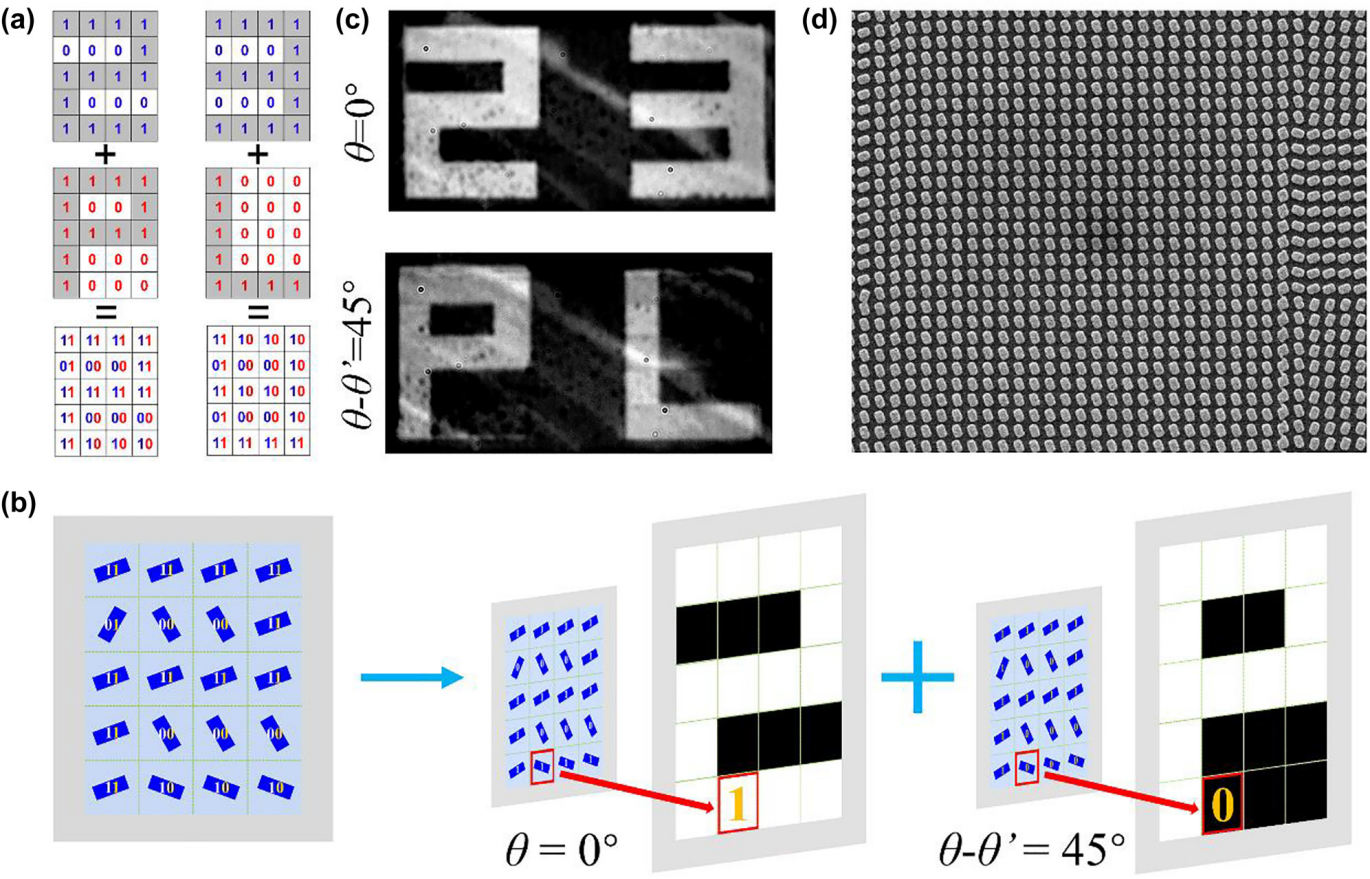
Schematic and experimental demonstration of the polarization encoded display metasurface. (a) The design approach of polarization encoded display. (b) The selection principle of orientation angles of nanorods for polarization encoded metasurface. (c) Measured optical images of polarization encoded metasurface at differently polarized incidences. (d) SEM image of polarization encoded metasurface composed of the unit cell structure of R1.

To confirm the performance of polarization encoded display, we design an encoded display metasurface under different linearly polarized illuminations. Figure 3a shows the encoded approach which combines two switched nanoprinting images displayed through a single structural rod R1 on a monolayer metasurface by modulating the polarization states of illumination, for example, each pixel of an image of number “23” and another image of letter “PL” (the top and middle panels of Figure 3a, respectively) can be marked as “1” and others marked as “0”, so the pixels of the combined metasurface consists of the coding patterns of “11”, “10”, “01”, and “00”, which corresponding to different orientation angles of nanorods. As shown in Figure 3b, it can choose different orientation angles of rods to constitute the polarization encoded display metasurface. The measured optical images under two linearly polarized illuminations with *θ* = 0° and *θ*-*θ’* = 45° are shown in Figure 3c, which are consistent with the simulation results (see Figure S2 in Supplemental Material). Figure 3d shows the SEM image of the polarization encoded display metasurface.

**Figure 4:**
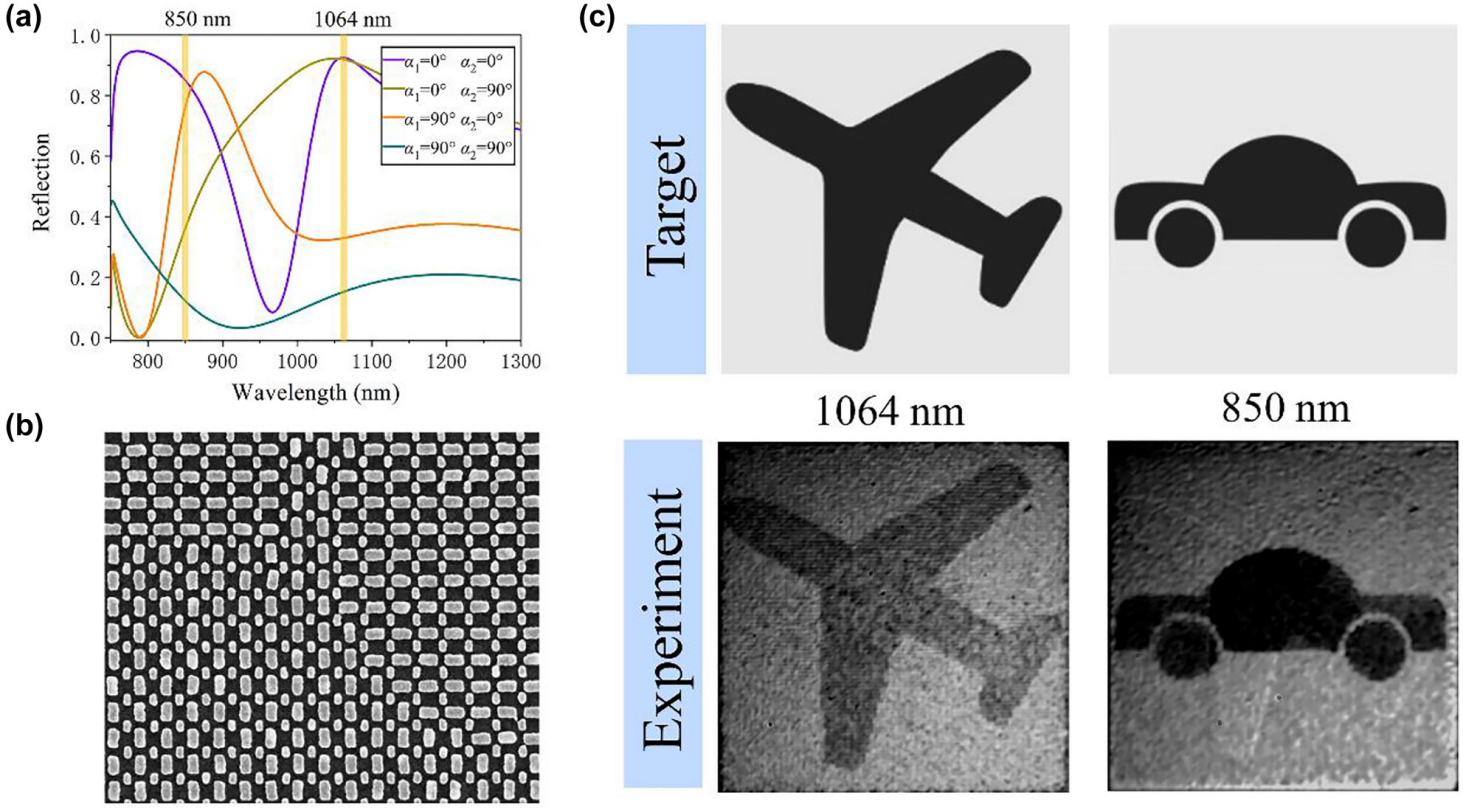
Wavelength encoded display metasurface. (a) The simulated reflection spectra of the supercells with different orientation angles. (b) SEM image of wavelength encoded metasurface composed of R1 and R2. (c) Target images and experimental results of wavelength encoded metasurface at 1064 nm and 850 nm incidences, respectively.

To enhance the information capacity of the metasurface, the wavelength encoded method can be introduced to increase the channels for nanoprinting display. Herein, we demonstrate a wavelength-dependent metasurface with switchable nanoprinting displays fewer than two linearly polarized illuminations of 850 nm and 1064 nm. Figure 4 shows the experimental results of the wavelength encoded display metasurface. Figure 4a shows the simulated reflection intensity of a supercell metasurface consisting of two Ag nanorods with different orientation angles, the results indicate that the additional reflection from a set of same-oriented nanorods has no obvious difference in reflection contrast from another set of rods under the operating wavelength, as compared with the metasurfaces consisting of the single-nanorod unit cell shown in Figure 2f, attributed to the fact that the coupling effect can be ignored between two orientation sets of rods. The supercell metasurface consisting of two kinds of nanorods (R1 and R2) was successfully fabricated, with an SEM image shown in Figure 4b. As a fact, little research has been reported to use wavelength to accomplish multifunctional grayscale imaging by a supercell metasurface. Here, we choose two target images where one presents a car and the other is an airplane (Figure 4c top panel) for 850 nm and 1064 nm, respectively, and the measured images (Figure 4c bottom panel) match the simulation results as shown in Figure S3.

**Figure 5:**
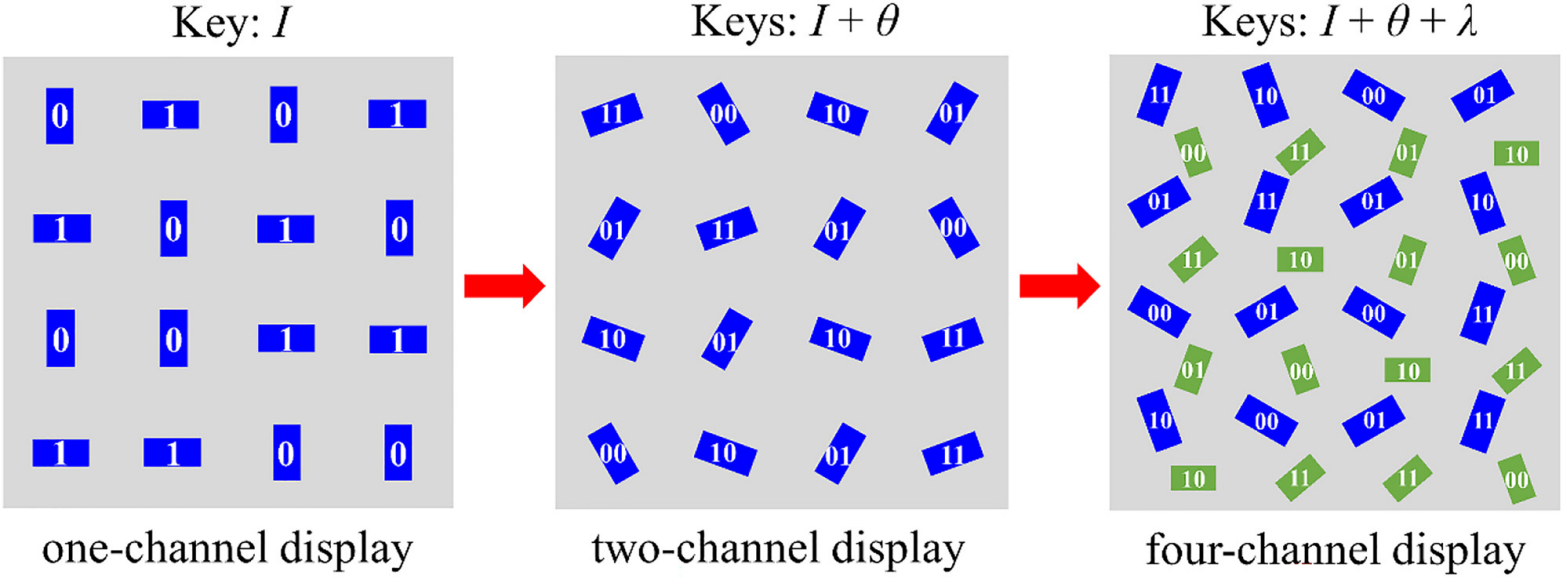
Design principle of four-channel display metasurface and the corresponding intensity, polarization, and wavelength encoded profiles.

To increase the information storage capacities, we designed a highly integrated four-channel display metasurface controlled by optical parameters, including intensity, polarization, and wavelength. Figure 5 shows the design principle of the four-channel metasurface, which demonstrates the arrangement of nanorods based on different encoding methods of optical parameters. More design details about the four-channel display metasurface are provided in Figure S4 and the design flowchart is shown in Figure S5 (in Supplemental Material).

**Figure 6:**
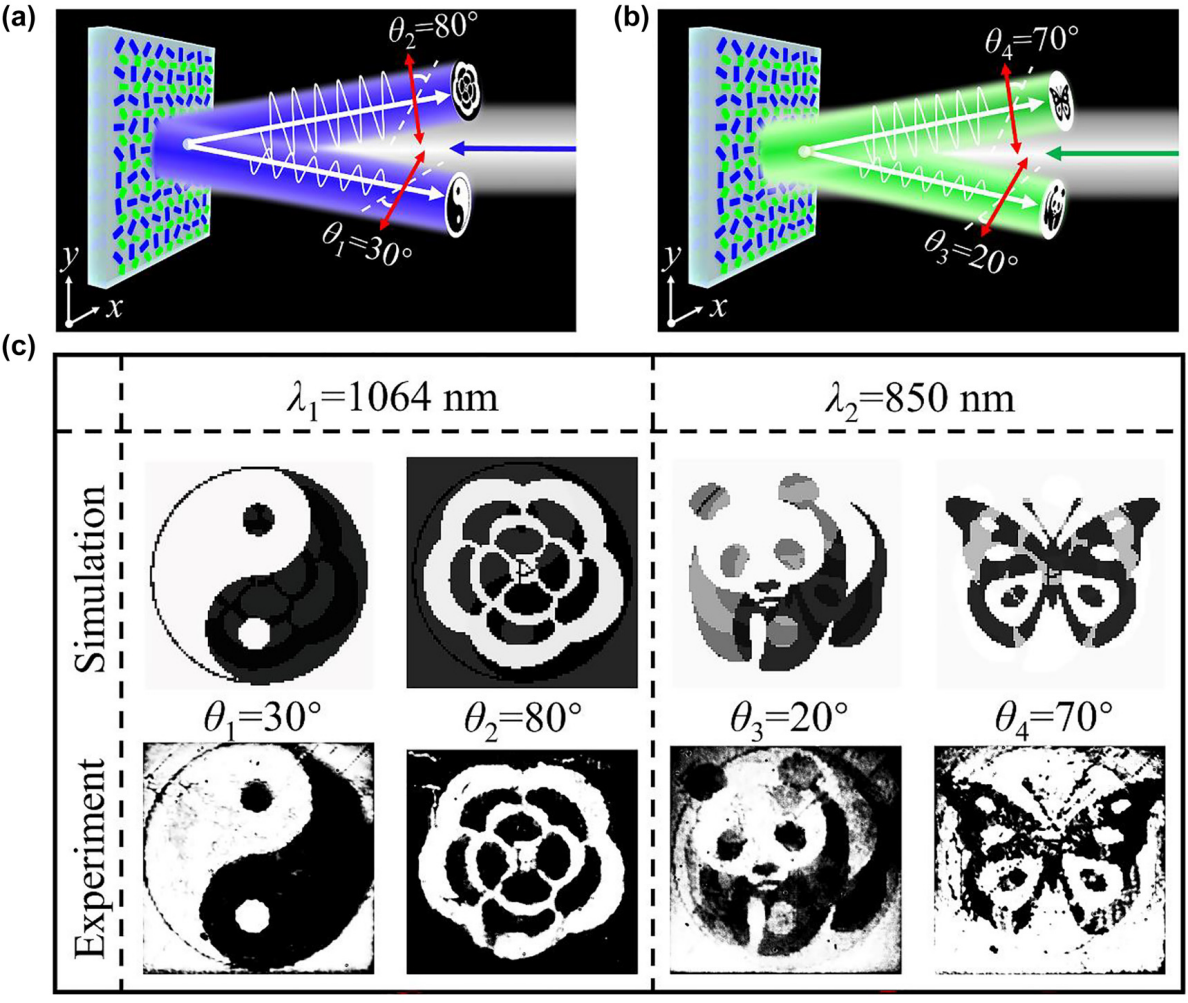
Schematic and characterization of four-channel display metasurface. (a, b) Schematic of four-channel display metasurface. (c–f) Measured optical four-channel images.

Figure 6 shows the nanoprinting display results that are experimentally captured at different polarization angles of 30°/80° and 20°/70° under the incident wavelength of 1064 nm and 850 nm, respectively. As Figure 6a shows, when a linearly polarized beam normally illuminates with the wavelength of 1064 nm, it can capture different nanoprinting images (‘Yin-Yang Fish’ and ‘Petal’) from two polarization channels of *θ*_1_ = 30° and *θ*_2_ = 80°. Meanwhile, when the incident wavelength is 850 nm, ‘Panda’ and ‘Butterfly’ are captured at different polarization channels of *θ*_3_ = 20° and *θ*_4_ = 70°, respectively, as shown in Figure 6b. With this principle, we fabricated four-channel display metasurface using standard electron beam lithography (EBL). The SEM image of the fabricated sample is shown in Figure S6 of Supplemental Material, which exhibits the well-fabricated supercell metasurface. The measurements of the nanoprinting images were captured by a common reflected optical device based on a home-built microscope, as shown in Figure S7. The simulation and measured optical nanoprinting images are shown in Figure 6c at different polarization angles under the incident wavelengths of 1064 nm and 850 nm. It obviously demonstrates that the encoded nanoprinting images are clear and with significant picture quality at two polarization channels of *θ*_1_ = 30° and *θ*_2_ = 80° under an incident wavelength of 1064 nm. Moreover, the second set of encoded nanoprinting images of ‘Panda’ and ‘Butterfly’ were captured from polarization channels of *θ*_3_ = 20° and *θ*_4_ = 70° at 850 nm, which to some extent did not keep the same high fidelity. The reason is that the difference in reflection intensities of R1 and R2 is only about 30% at 850 nm, and thus results in some noise. Due to the limitation of the experiment, we have chosen one of the wavelength channels of 850 nm as the incident wavelength. As the simulation results are shown in Figure S1, if the incident wavelength of 780 nm were used, the difference in reflection intensity at two-wavelength channels can reach 70%, which will decrease the noise and eliminate crosstalk between nanoprinting images from two-wavelength channels. Here, we compare the simulation results of polarization encoded images with the polarization angles of *θ*_3_ = 20° and *θ*_4_ = 70° under 850 nm and 780 nm, as shown in Figure S8 in Supplemental Material. The simulation results show that the nanoprinting images under the incident wavelength of 780 nm with high fidelity and low crosstalk can be obtained in practical applications. Hence, simulation and experimental results validate that our proposed four-channels encoded display metasurfaces can significantly enhance the information capacity and improve the security of optical anticounterfeiting.

## 3 Conclusions

In conclusion, we proposed and demonstrated a novel four-channel subwavelength nanoprinting metasurface that can display four different encryption images by optical switching parameters as their respective security keys. Specifically, a monolayer metasurface was successfully designed, which can manipulate the reflection amplitude by rotating the orientation angle of the nanorod. In addition, based on Malus’s law, polarization can be regarded as a degree of freedom to multichannel display. More importantly, we carefully designed an ultracompact supercell consisting of two kinds of nanorods with different operating wavelengths as another optical degree of freedom for multichannel display. To verify these ideas, the simulation and experimental results demonstrated that the metasurface can separately display two easily recognized images. Finally, a four-channel metasurface was designed and fabricated, which can be considered as a novel strategy for encryption information. Compared with other encryption metasurfaces, our four-channel display metasurface is the first reported design of four independent channels and has a simplified imaging system that can capture all images in the near-field. Besides, it has advantages such as high storage capacity, ultracompactness, multichannel/multifunction, lightweight, and cost-cutting. The proposed encoded display metasurface will provide a great convenience for potential applications of advanced anticounterfeiting technology.

## 4 Experimental section

### 4.1 Numerical characterizations

To obtain the reflection amplitudes in dependence of nanorod size, the length (*l*), width (*w*), and heights (*h*) were swept in ranges of 150–350 nm, 80–150 nm, and 50–150 nm, respectively, while the period was kept as 520 nm to reasonably arrange supercell metasurface. The reflection results were calculated by the computer simulation technology (CST) Microwave Studio software.

### 4.2 Sample preparation

All metasurfaces were fabricated on the glass (SiO_2_) substrates. First, the substrates were cleaned with acetone solution, ethyl alcohol solution, and purified water for 15 min under ultrasonic conditions in sequence. A polymethylmethacrylate (PMMA) resist layer was amorphously spin-coated onto the substrate, which was heated on a hot plate at 180 °C for 2 min. Then, the processed substrates were etched by electron beam lithography to produce the designed structure. Next, an amorphous layer of 100 nm thick of Ag film was deposited using electron beam evaporation. Last, the samples were performed in tepid acetone at 60 °C until the redundant Ag films were peeled off.

### 4.3 Optical measurement

The nanoprinting images were measured by a home-built microscope as shown in Figure S7. A collimated broadband light beam was emitted by a halogen lamp and a collimator, a bandpass filter, and a linear polarizer (LP) were used to prepare and select the desired linearly polarized beam. Then, the linearly polarized beam was concentrated on the surface of the sample by the lens 1 with a 10 cm focal length and a 100 × objective, where the beam splitter prism (BSP) was used to separate the incident and reflected light beams. Last, the nanoprinting images reflected by the samples were captured by a charge-coupled device (CCD) camera, where the focal length of lens 2 is 10 cm.

## Supplementary Material

Supplementary Material Details
