# Supplementary material for: Four-channel display and encryption by near-field reflection on nanoprinting metasurface
Source: Nanophotonics. 2022 Jun 14;11(14):3365–74. doi: 10.1515/nanoph-2022-0216 (PMC11501998; doi:10.1515/nanoph-2022-0216)
Supplement: Supplementary file 1 — Supplementary Material Details [file j_nanoph-2022-0216_suppl.docx]

**Supplemental Material**

Four-Channel Display and Encryption by Near-Field Reflection on Nanoprinting Metasurface

*Yue Cao*^1,2,3^*, Lili Tang*^1^*, Jiaqi Li*^1^*, Chengkuo Lee*^2,3^*^*^, and Zheng-Gao Dong*^1^*^*^*

**^1^**School of Physics, Southeast University, Nanjing 211189, China

**^2^** Department of Electrical and Computer Engineering, National University of Singapore, Singapore 117583, Singapore

**^3^** Center for Intelligent Sensors and MEMS(CISM), National University of Singapore, Singapore 117542, Singapore

**Corresponding authors:**

Zheng-Gao Dong, School of Physics, Southeast University, Nanjing 211189, China

E-mail: [zgdong@seu.edu.cn](mailto:zgdong@seu.edu.cn)

Chengkuo Lee, Department of Electrical and Computer Engineering, National University of Singapore, Singapore 117583, Singapore

E-mail: [elelc@nus.edu.sg](mailto:elelc@nus.edu.sg)

**Note S1**

**The principle analysis of amplitude modulation**

For a nanorod with an orientation angle *α* relative to the reference coordinate system, the Jones matrix is written as

| $J_{\text{rod}}=R(-\alpha)T_{0}R(\alpha)$ | S1 |
| --- | --- |

where R(*α*) is the rotation matrix, *T*_0_ is the transmission matrix.

| $R(\alpha)\text{=}\left[\begin{matrix} \cos\alpha& \sin\alpha\\ -\sin\alpha& \cos\alpha\end{matrix}\right]$ | $R(-\alpha)\text{=}\left[\begin{matrix} \cos\alpha& -\sin\alpha\\ \sin\alpha& \cos\alpha\end{matrix}\right]$ | $T_{0}\text{=}\left[\begin{matrix} A & 0 \\ 0 & B \end{matrix}\right]$ | S2 |
| --- | --- | --- | --- |

where $\alpha$ is the orientation angle of the nanorod, $A$ and $B$ are the complex reflection coefficients of the nanostructure along its fast and slow axes, respectively.

When a linearly polarized (LP) beam with the polarization direction $\theta$ vertically illuminates the nanorod, the Jone matrix of the reflected beam can be written as

| $J_{\text{rod}}=\left[\begin{matrix} \cos\alpha& -\sin\alpha\\ \sin\alpha& \cos\alpha\end{matrix}\right]\left[\begin{matrix} A & 0 \\ 0 & B \end{matrix}\right]\left[\begin{matrix} \cos\alpha& \sin\alpha\\ -\sin\alpha& \cos\alpha\end{matrix}\right]\left[\begin{matrix} \cos\theta\\ \sin\theta\end{matrix}\right]$ | S3 |
| --- | --- |
| $=\left[\begin{matrix} A\cos(\alpha-\theta)\cos(\alpha)+B\sin(\alpha-\theta)\sin\alpha\\ A\cos(\alpha-\theta)\sin(\alpha)-B\sin(\alpha-\theta)\cos\alpha\end{matrix}\right]$ |

If the intensity of the incident beam is *I*_0_, the intensity *I* of reflected light can be expressed as

| $I=I_{0}[A^{2}\cos^{2}(\alpha-\theta)+B^{2}\sin^{2}(\alpha-\theta)]$ | S4 |
| --- | --- |

The proposed nanorods that effectively manipulate the co-polarization reflection intensity can be regarded as an ideal polarizer for its long axis and short axis. So, we can set as $A=1$ and $B=0$, the reflection intensity *I* is deduced as

| $I=I_{0}\cos^{2}(\alpha-\theta)$ | S5 |
| --- | --- |

**Note S2**

**The optical response of three sized nanorods with different parameters**


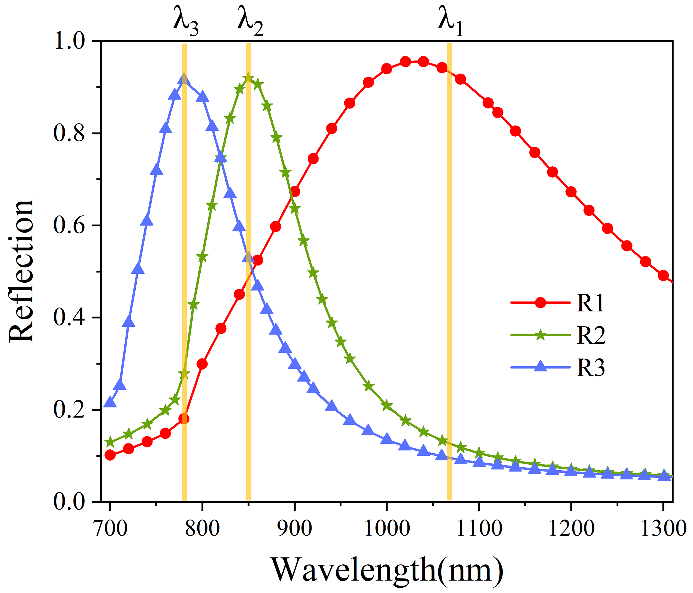


**Figure S1.** Simulated reflection spectra for three sizes of nanorods (R1, R2, and R3) under different incident wavelengths (*λ*_1_=1064 nm, *λ*_2_=850 nm, and *λ*_3_=780 nm).

From Figure 2 (f), we can find that the reflection intensity of R1 has reached 0.5 at 850 nm, and thus could cause some crosstalk for two-channel wavelength encoded display metasurface. To reduce the crosstalk, we designed the R3 structure whose reflection intensity exceeds 0.9 at 780 nm, and the reflection intensity of R1 is less than 0.2 at 780 nm, which shows the crosstalk of wavelength channels chosen 1064 nm and 780 nm is small enough to be theoretically omitted. The parameters of the R3 structure are length *l*_3_=180 nm, width *w*_3_=100 nm, height *h*_3_=100 nm, and the period remains constant (p=520 nm).

**Note S3**

**The simulation images of polarization and wavelength encoded metasurfaces**


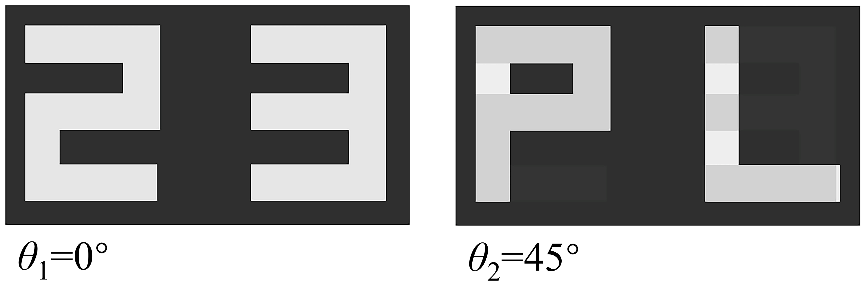


**Figure S2.** Simulated results at different polarization angles of *θ*_1_=0° and *θ*_2_=45° under the incident wavelength of 1064 nm.


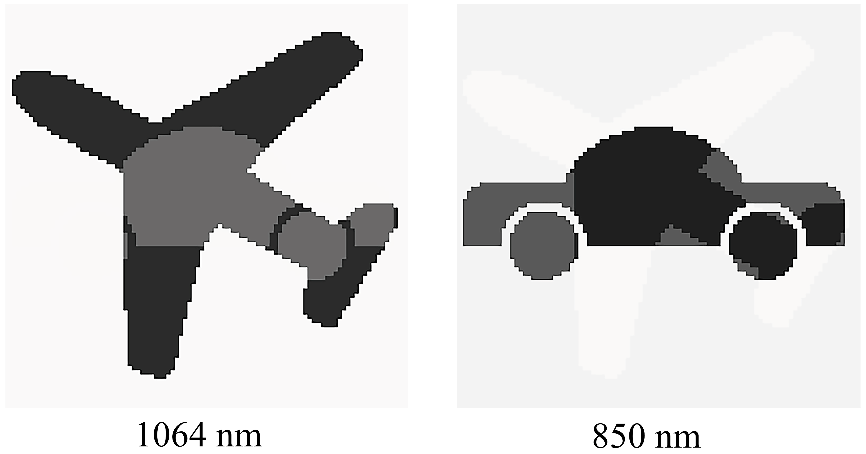


**Figure S3.** Simulated results at incident wavelengths of 1064 nm and 850 nm.

**Note S4**

**Optical manipulation parameters of multichannel encoded display metasurface**


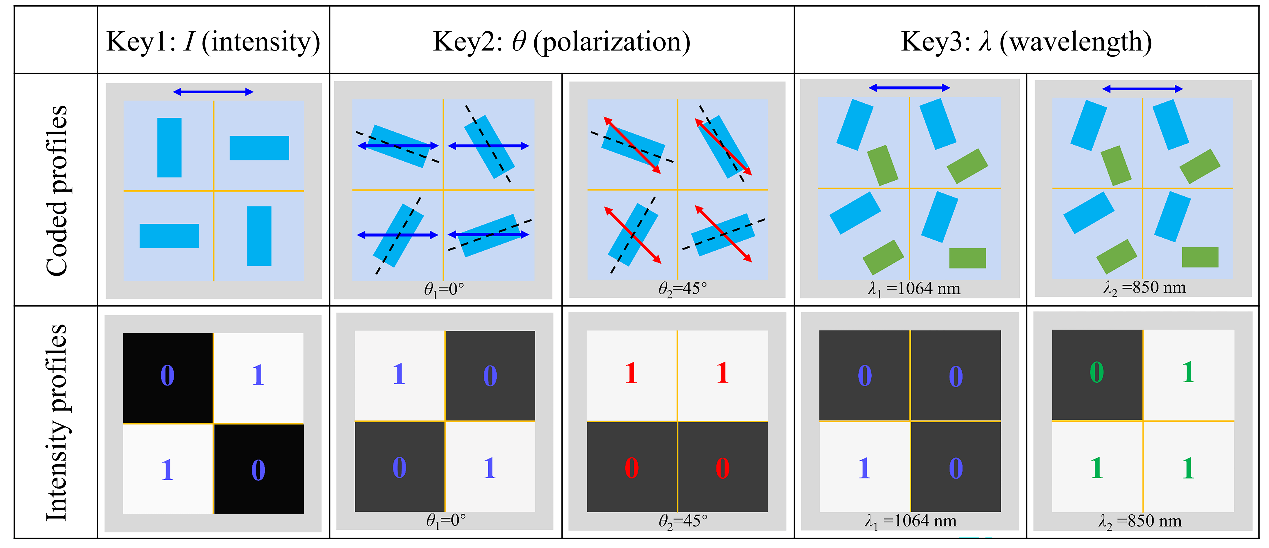


**Figure S4.** Encoded diagram of optical parameters of (intensity, polarization, and wavelength) multichannel display metasurface.


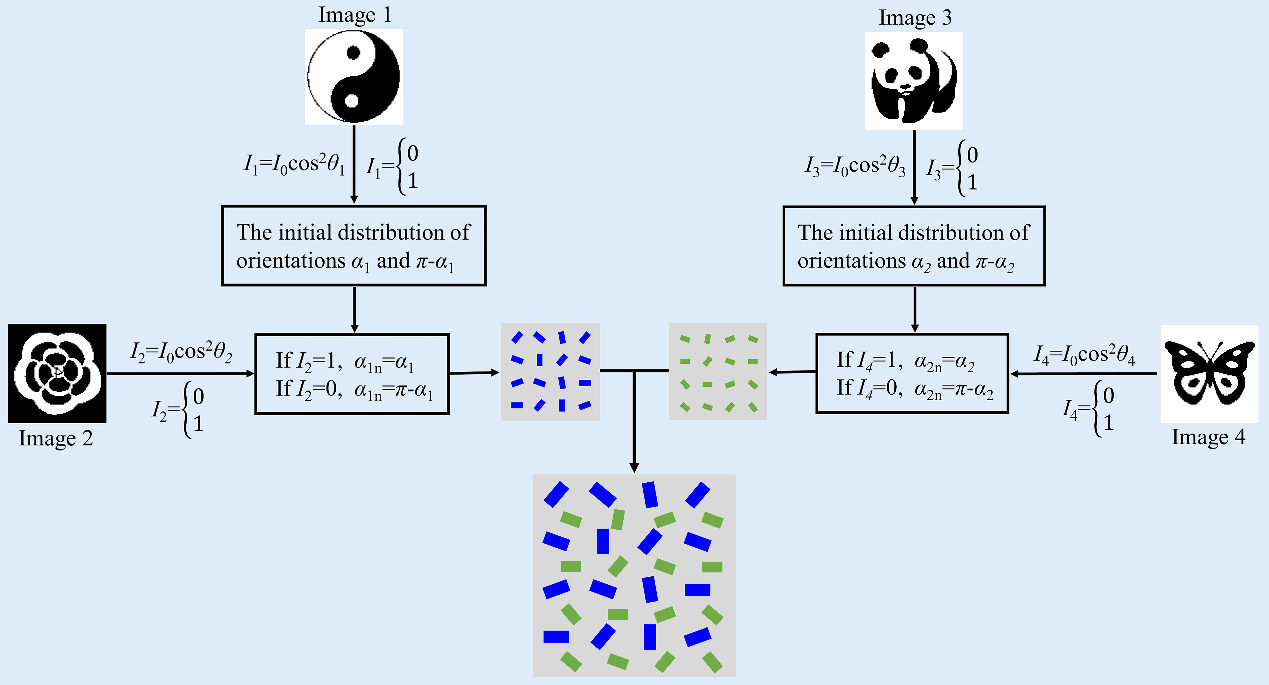


**Figure S5.** Design flowchart of four-channel encoded display metasurface.

To elaborate the design process of the proposed metasurface, a flowchart of four-channel encoded display metasurface is shown in Figure S5, which is utilized for integrating four independent channels into an ultracompact display metasurface. Firstly, according to the simulation results and the intensity modulation principle of Malus’s law, we can confirm four polarization channels (*θ*_1_, *θ*_2_, *θ*_3_, and *θ*_4_) under two wavelength channels (*λ*_1_ and *λ*_2_). Then, the initial distributions of the orientation angle of Image 1 and Image 3 (*α*_1_ and *α*_2_) can be calculated, where all orientation angles of nanorod distribute from 0 to *π* reflection intensity corresponds to two orientation angles. Next, the orientation distributions of Image 2 and Image 4 can be extracted, which also lie in the interval [0, *π*] and satisfy Malus’s law. Further, we can judge the intensity values of Image 2 and Image 4 (*I*_2_ and *I*_4_), if the intensity value of *I*_2_ (or *I*_4_) is 1(a “high” value), the corresponding initial orientation angle is *α*_1_ (or *α*_2_), on the contrary, the initial orientation angle is *π-α*_1_ (or *π-α*_2_). Therefore, we can confirm the final distributions of orientation angle *α*_1n_ (or *α*_2n_) by the above judging operation. After all judging operation cell by cell, we finally obtain all the distributions of orientation angle for the four-channel display metasurface.


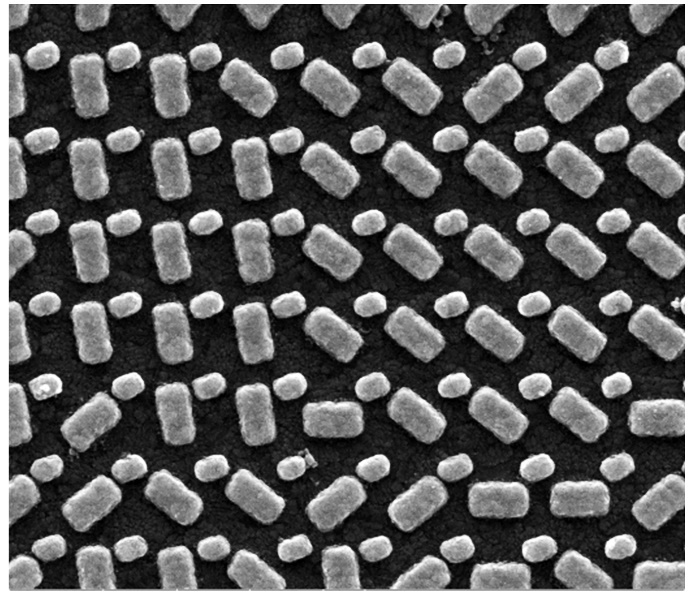


**Figure S6.** SEM image of four-channel display metasurface consisting of R1 and R2.


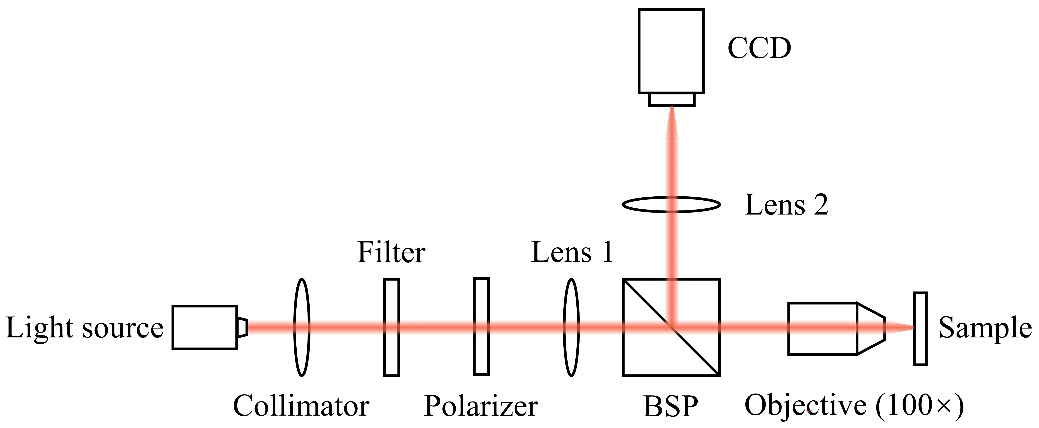


**Figure S7.** Experimental setup for measuring the display images. The incident light beam is generated by a broadband halogen lamp. BSP: beam splitter prism.


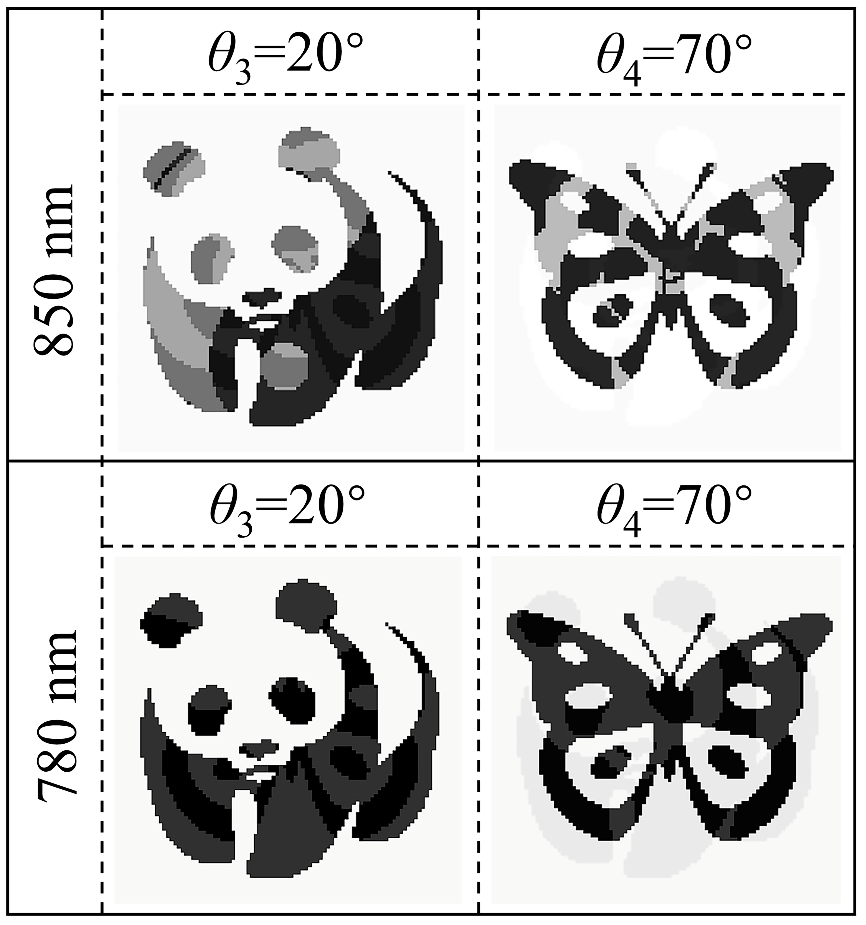


**Figure S8.** Simulation results of polarization encoded images under 850 nm and 780 nm with *θ*_3_=20° and *θ*_4_=70°.
